# Role of sequence and position of the cleavage sites in prothrombin activation

**DOI:** 10.1016/j.jbc.2021.100955

**Published:** 2021-07-12

**Authors:** Bosko M. Stojanovski, Enrico Di Cera

**Affiliations:** Edward A. Doisy Department of Biochemistry and Molecular Biology, Saint Louis University School of Medicine, St Louis, Missouri, USA

**Keywords:** prothrombin, thrombin, serine protease, single-molecule biophysics, protein conformation

## Abstract

In the penultimate step of the coagulation cascade, the multidomain vitamin-K-dependent zymogen prothrombin is converted to thrombin by the prothrombinase complex composed of factor Xa, cofactor Va, and phospholipids. Activation of prothrombin requires cleavage at two residues, R271 and R320, along two possible pathways generating either the intermediate prethrombin-2 (following initial cleavage at R271) or meizothrombin (following initial cleavage at R320). The former pathway is preferred in the absence of and the latter in the presence of cofactor Va. Several mechanisms have been proposed to explain this preference, but the role of the sequence and position of the sites of cleavage has not been thoroughly investigated. In this study, we engineered constructs where the sequences ^261^DEDSDRAIEGRTATSEYQT^279^ and ^310^RELLESYIDGRIVEGSDAE^328^ were swapped between the R271 and R320 sites. We found that in the absence of cofactor Va, the wild-type sequence at the R271 site is cleaved preferentially regardless of its position at the R271 or R320 site, whereas in the presence of cofactor Va, the R320 site is cleaved preferentially regardless of its sequence. Additional single-molecule FRET measurements revealed that the environment of R271 changes significantly upon cleavage at R320 due to the conformational transition from the closed form of prothrombin to the open form of meizothrombin. Detailed kinetics of cleavage at the R271 site were monitored by a newly developed assay based on loss of FRET. These findings show how sequence and position of the cleavage sites at R271 and R320 dictate the preferred pathway of prothrombin activation.

Prothrombin is a multidomain vitamin-K-dependent zymogen that is converted to the active enzyme thrombin in the penultimate step of the coagulation cascade (1). The multidomain assembly comprises an N-terminal Gla domain (residues 1–46), kringle 1 (residues 65–143), kringle 2 (residues 170–248), and a C-terminal protease domain (residues 285–579) connected by three intervening linkers (Fig. 1*A*) (2, 3, 4, 5). Thrombin formation requires cleavage of prothrombin at two sites, R271 and R320, by the prothrombinase complex composed of factor Xa (fXa), cofactor Va (fVa), and phospholipids (6, 7, 8, 9). Cleavage at R271 sheds the Gla domain and two kringles and generates the inactive intermediate prethrombin-2 (Fig. 1*A*). The alternative cleavage at R320 separates the A and B chains of the protease domain that remain connected through a disulfide bond and generates the active intermediate meizothrombin (Fig. 1*A*). The presence of fVa directs activation along the meizothrombin pathway and greatly (>1000-fold) accelerates the rate of cleavage at R320, but has a smaller effect (30-fold) on the cleavage of meizothrombin at R271 (9). Meizothrombin accumulates as an intermediate when prothrombinase is assembled on the membrane of red blood cells (10), synthetic liposomes (6, 9, 11, 12), and microparticles (13). On the other hand, platelets direct prothrombinase to cleave initially at R271 along the prethrombin-2 pathway (14, 15), which is also preferred in the absence of fVa (6, 7).

**Figure 1:**
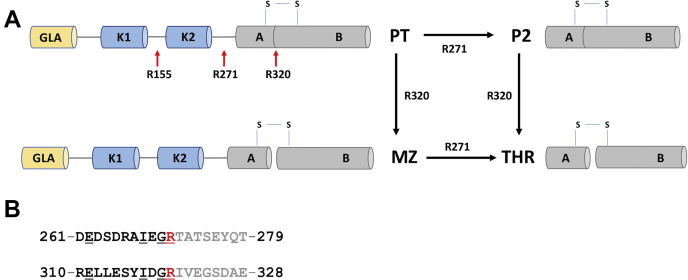
**Scheme of prothrombin activation.***A*, schematic representation of the structural architecture of prothrombin (PT) comprised of the γ-carboxyglutamate (GLA) and two kringles (K) domains that constitute fragment 1.2 and a protease domain composed of A and B chains. Activation of prothrombin proceeds along two mutually exclusive pathways where initial cleavage at R320 or R271 leads to formation of meizothrombin (MZ) or prethrombin-2 (P2), respectively, before producing the final product thrombin (THR). Under certain conditions, initial cleavage at R155 generates the prethrombin-1 intermediate composed of kringle 2 and the protease domain. *B*, alignment of the P11–P8′ residues comprising the R271 and R320 cleavage sites (*red*). Residues in the primed region of the sequence (C-terminal to the P1 Arg) are shown in *gray*, while nonprimed residues (N-terminal to the P1 Arg) are shown in *black*. Conserved amino acids are *underlined*.

Although meizothrombin and prothrombin share the same multidomain assembly, there are significant differences in the structure of the two proteins. Cleavage at R320 triggers the Huber–Bode mechanism of zymogen activation (16) where a new N-terminus engages a conserved Asp residue next to the catalytic Ser and organizes the architecture of the active site and primary specificity pocket (17, 18, 19), along with exosite I (20), the Na^+^-binding site (19, 21), and the autolysis loop (22). Activation also shifts the predominant closed conformation of prothrombin to the open form of meizothrombin (2, 23, 24, 25). The shift affects cleavage at R271 by either fXa or prothrombinase, which is considerably faster in meizothrombin (8, 9). Prothrombin mutants stabilized in the open form are activated by prothrombinase with accumulation of the prethrombin-2 intermediate because of increased specificity for the R271 site (2). Binding of substrates to the active site of prothrombin has been reported to enhance cleavage at R271 (26, 27, 28), presumably because active site occupancy stabilizes the open form in both prothrombin and meizothrombin (2, 23, 24, 25). On the other hand, mutations that interfere with the zymogen-to-protease transition and the concomitant shift from the closed to the open form (22) decrease the rate of cleavage at R271 (26).

Because fXa preferentially cleaves at R271 and prothrombinase preferentially cleaves at R320 (8, 9), it is important to establish whether the preference is determined by the conformation of the protein and/or the specific sequence around the cleavage sites at R271 and R320. About 79% of residues in the P11–P8′ positions (29) of the ^261^DEDSDRAIEGRTATSEYQT^279^ and ^310^RELLESYIDGRIVEGSDAE^328^ sequences are not conserved between the two cleavage sites (Fig. 1*B*), especially in the primed region (C-terminally of the P1 Arg). In the nonprimed region (N-terminally of the P1 Arg), four out of 11 residues are conserved at both cleavage sites, including the P1 Arg, P2 Gly, P4 Ile, and P10 Glu (Fig. 1*B*). Previous studies have addressed how the sequence affects cleavage at R320 and shown that replacements at the P1–P3 positions in prethrombin-2 adversely affect formation of thrombin (30). However, the P1 and P2 residues are conserved between the R271 and R320 sites, and the P3 residue is acidic in both cases (Fig. 1*B*). The P1′–P3′ residues at the R320 site have also been mutated and shown to have a very small effect on the activation rate (26). Overall, these previous studies have offered little insight into the molecular origin of preferential cleavage at the R271 and R320 sites. Differences in the extended sequences at the two sites turned out to be more informative, as shown below.

## Results

### Effect of the sequence at the R271 and R320 sites on the pathway of activation

**Figure 2:**
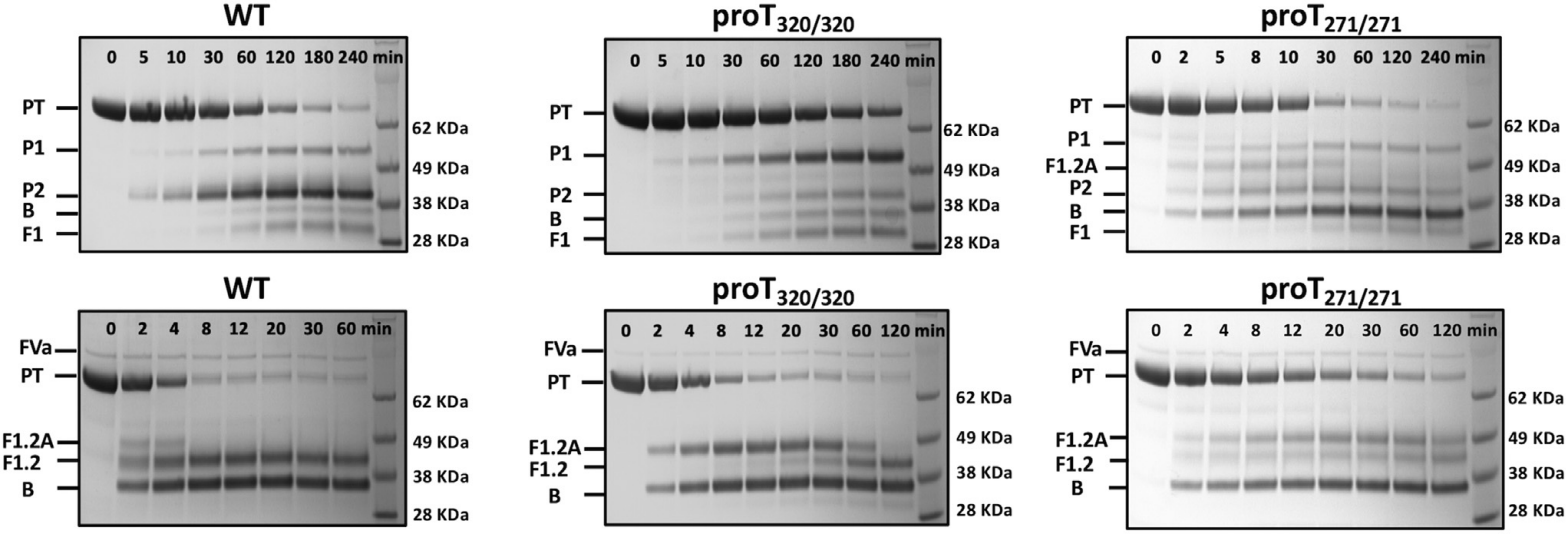
**Kinetics of prothrombin activation.** Activation of prothrombin wild-type and mutants monitored by SDS-PAGE under reducing conditions either in the absence (*upper panels*) or presence (*lower panels*) of 30 nM fVa. The concentration of fXa was 0.2 nM or 4 nM in the presence or absence of fVa, respectively. The identity of all bands was determined by N-terminal sequencing. Abbreviations are: PT (prothrombin), P1 (prethrombin-1), P2 (prethrombin-2), A and B chain of the protease domain, F (fragment). The F1, F1.2, and F1.2.A fragments denote the N-terminal domains of prothrombin retained after cleavage at R155 (F1), R271 (F1.2), and R320 (F1.2.A) (see also Fig. 1). Optical densitometry was used to quantify the accumulation of various intermediates as shown in Figure 3. Experimental conditions are: 20 mM Tris, 145 mM NaCl, 5 mM CaCl_2_, 60 μM phospholipids, 60 μM DAPA, 0.1% PEG8000, pH 7.5 at 25 °C.

**Figure 3:**
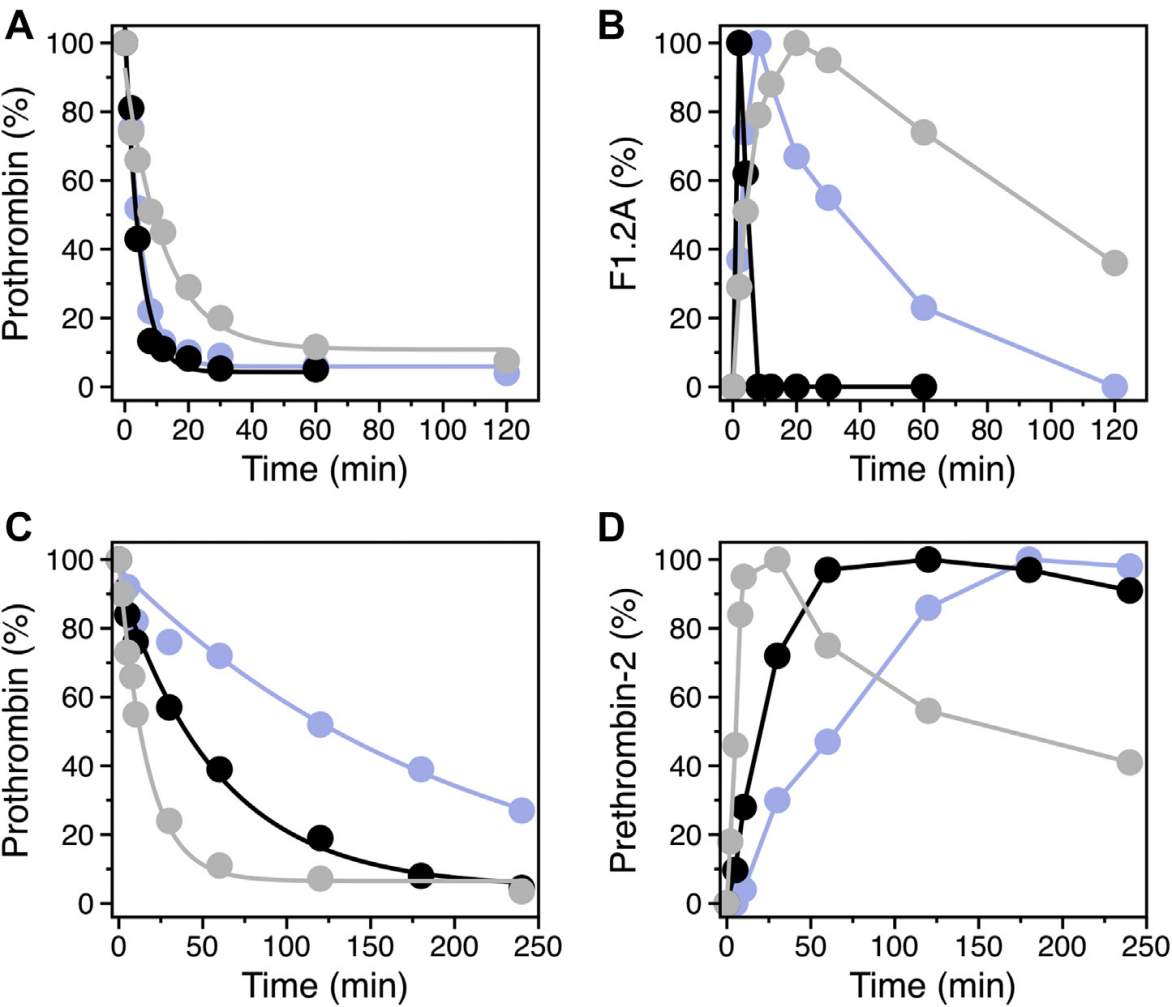
**Intermediates of prothrombin activation.** Optical densitometry of the SDS-PAGE gels shown in Figure 2 using ImageJ quantifies the intensities of the bands of (*A*) prothrombin and (*B*) F1.2 A in the presence of fVa, and of (*C*) prothrombin and (*D*) prethrombin-2 in the absence of fVa. Data points refer to wild-type (*black*), proT_320/320_ (*purple*), and proT_271/271_ (*gray*). Accumulation of F1.2 A (*B*) for activation of proT_320/320_ and proT_271/271_ by prothrombinase is due to slower cleavage at R271. The slower consumption of prothrombin (*C*) and the delayed appearance of prethrombin-2 (*D*) for activation of proT_320/320_ by fXa are also due to slower cleavage at R271.

The prothrombin loop-swap mutants proT_320/320_ and proT_271/271_ were engineered to examine how the pathway of activation is affected by the amino acid sequence at the R271 and R320 cleavage sites. proT_320/320_ has the entire P11–P8′ sequence at the R271 site replaced with that of the R320 site and vice versa for proT_271/271_ (Fig. 1*B*). Prothrombin is activated by fXa in the absence of fVa with initial cleavage at R271 and formation of prethrombin-2, followed by cleavage at R320 and appearance of thrombin (Fig. 2). The lack of detectable levels of meizothrombin indicates that the two scissile bonds are attacked sequentially, with the proteolysis at R271 always preceding that at R320. If the sequential cleavage is caused by differences in the amino acid sequence at the two sites of prothrombin, then replacing the sequence at the R320 site with that of the R271 site should accelerate cleavage by fXa at the swapped region in proT_271/271_ and give evidence of activation also along the meizothrombin pathway. Indeed, proT_271/271_ is activated by fXa along both pathways. Activation along the meizothrombin pathway is evident from the appearance of the fragment 1.2.A band during the early time course of the reaction, which denotes initial cleavage at R320, while accumulation of prethrombin-2 documents the additional cleavage of proT_271/271_ at the R271 site (Fig. 2). Characterization of the activation pathway of proT_320/320_ provides further evidence that the sequence makes an important contribution in directing the sequential cleavage by fXa. proT_320/320_ is activated by fXa along the prethrombin-2 pathway, but the initial cleavage at R271 is significantly slower and the appearance of the prethrombin-2 band is delayed compared with wild-type (Figs. 2 and 3). The slower rate of cleavage implies that fXa has a strong preference for the native sequence at the R271 site. Initial cleavage at R155 leads to formation of the zymogen prethrombin-1 composed of the kringle 2 and protease domain (Fig. 1*A*). The prethrombin-1 band was also detected for all prothrombin variants, with the intensity being most pronounced for proT_320/320_ (Fig. 2) due to slower cleavage at the 271 site. The appearance of prethrombin-1 is not due to cleavage by thrombin because the reactions were carried out in the presence of DAPA (a selective thrombin inhibitor). Furthermore, formation of prethrombin-1 was also detected with the inactive prothrombin mutant S525A, where the catalytic Ser is replaced by Ala, proving that cleavage at R155 is catalyzed by fXa (5).

Next, we examined the pathway of activation in the presence of fVa. The prothrombinase complex activates prothrombin along the meizothrombin pathway documented by the transient appearance of fragment F1.2.A during the early stages of the reaction (Figs. 2 and 3). Disappearance of this band signals subsequent cleavage at R271 and formation of thrombin (Fig. 2). If sequential cleavage is caused by differences in the amino acid sequence at the two sites of prothrombin, then replacing the sequence at the R271 site with that of the R320 site should accelerate cleavage by prothrombinase at the swapped site in proT_320/320_ and shift activation toward the prethrombin-2 pathway. Contrary to this expectation, prothrombinase activates proT_320/320_ along the meizothrombin pathway with no evidence of prethrombin-2 formation (Fig. 2). The rate of cleavage at the R271 site carrying the sequence of the R320 site is significantly slower, as evidenced by the prolonged accumulation of fragment F1.2.A and the delayed appearance of fragment F1.2 compared to wild-type (Figs. 2 and 3). Importantly, prothrombinase activates proT_271/271_ along the meizothrombin pathway as well, proving that swapping the R320 sequence with that of the R271 site does not change the pathway of activation (Fig. 2). The R271 site in proT_271/271_ is cleaved at a slower rate because of the prolonged accumulation of fragment F1.2.A compared with wild-type (Figs. 2 and 3). A similar observation has been reported for a prothrombin mutant with the P1′–P3′ residues at the R320 site replaced by those of the R271 site (26). The likely explanation is that replacement of the P1′–P3′ sites in this and the proT_271/271_ mutant impairs the transition to the open form of meizothrombin based on correct formation of the I321-D359 H-bond of the Huber–Bode mechanism of activation (16), thereby making the R271 site less susceptible to cleavage. Overall, our data indicate a major difference in preferential cleavage between fXa and prothrombinase, due entirely to the absence/presence of fVa. Preferential cleavage is dictated by the sequence and not the position in the absence of fVa and by the position but not the sequence in the presence of cofactor.

### FRET analysis of prothrombin activation by fXa

Current assays that monitor cleavage at R271 in prothrombin rely upon rates measured by optical densitometry or by monitoring modest differences in the level of DAPA fluorescence due to binding to meizothrombin and thrombin (9). To overcome these limitations and examine how the sequence affects the rate of cleavage at R271, we developed a FRET assay using the mutant S101C/R155A/S478C/S525A, proT_RS_, where the replacement R155A prevents cleavage at this site, S525A avoids (auto)proteolysis and the two Cys substitutions position an Alexa Fluor 555/647 pair at kringle 1 and the protease domain. Labeling at these sites has no influence on the functional properties of prothrombin (23).

**Figure 4:**
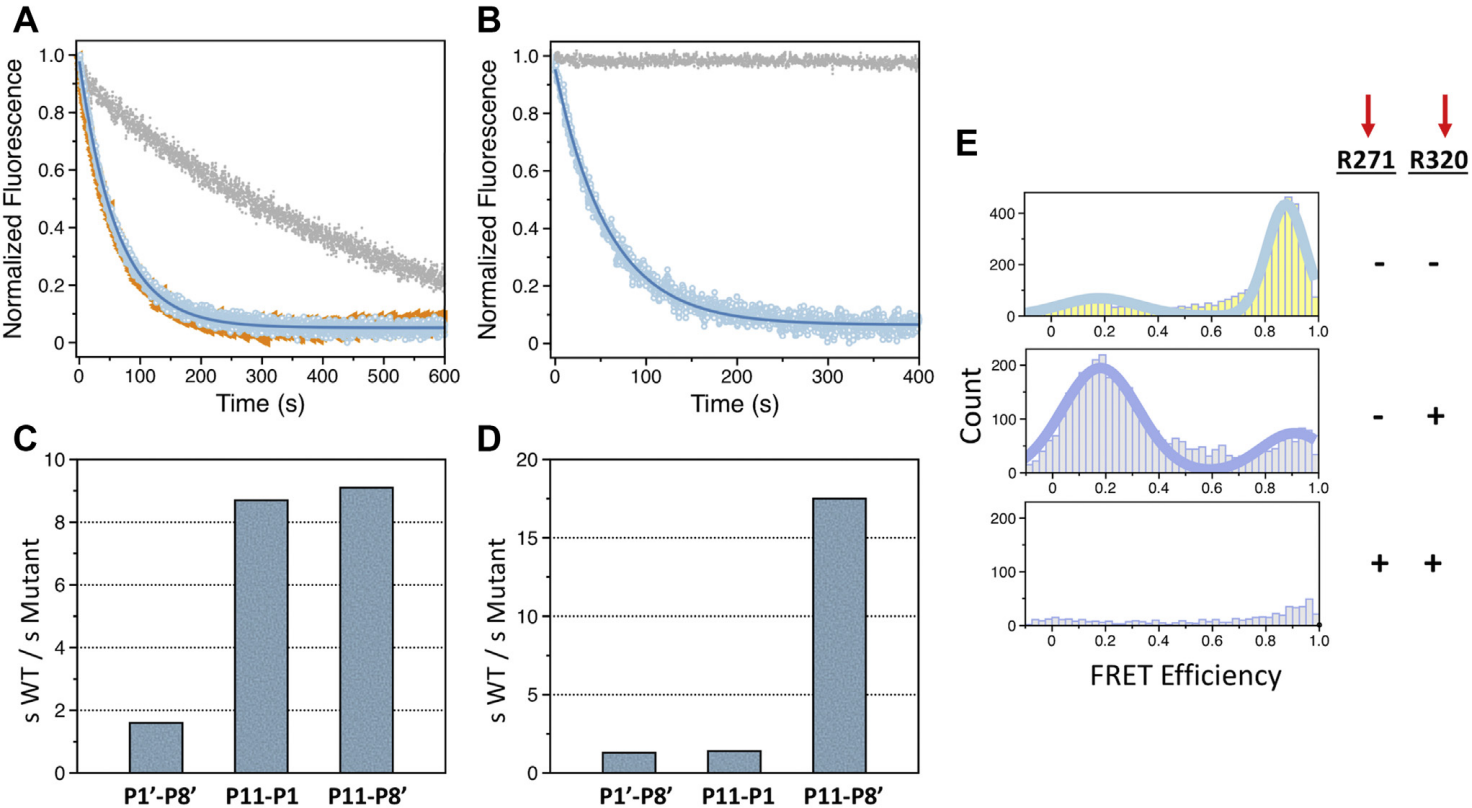
**FRET assay of prothrombin activation.** Progress curves for the activation of (*A*) prothrombin by fXa (20 nM) and (*B*) meizothrombin by prothrombinase (0.1 nM fXa, 10 nM fVa) monitored as loss of FRET. The FRET construct (S101C/R155A/S478C/S525A) is in *blue*, while those containing the R271Q and R320Q mutations are in *gray* and *orange*, respectively. Values of s = *k*_cat_/*K*_m_ for the reaction between (*C*) prothrombin and factor Xa and (*D*) meizothrombin and prothrombinase. Ratios were calculated by dividing the rate for the FRET mutant containing the wild-type 271 sequence over the indicated swapped sequence. The *k*_cat_/*K*_m_ values (Table 1) were obtained from the FRET assay. *E*, smFRET profiles of proT_RS_ labeled at the 101C/478C pair with the Alexa Fluor 555/647 fluorophores. Prothrombin (*yellow* histogram) and meizothrombin (*gray* histograms) predominantly populate closed and open conformations that are characterized by high and low FRET efficiencies, respectively. The R320 bond was selectively cleaved upon incubation with ecarin and subsequent cleavage at R271 was initiated with fXa. Cleavage of the respective bonds is indicated. Experimental conditions are listed under Experimental procedures.

When the activation of proT_RS_ by fXa was monitored by changes in FRET, the resulting progress curve could be described by a single exponential (Fig. 4*A*) with a rate constant of 0.53 μM^−1^s^−1^ in close agreement with the value of *k*_cat_/*K*_m_ = 0.64 μM^−1^s^−1^ reported for cleavage of R271 by fXa (9). The loss of FRET coincides with cleavage at R271 and separation of the protease domain from the rest of the protein containing the Gla domain and two kringles (Figs. 1*A* and 4*A*). To demonstrate this point, R271 and R320 were replaced individually by Gln. SDS-PAGE confirmed that fXa does not cleave at the R271Q and R320Q sites (data not shown). Progress curves of proT_RS,R320Q_ (cleaved at R271 only) activation by fXa measured by changes in FRET were superimposable to those of proT_RS_ (cleaved at both R271 and R320), but those of proT_RS,R271Q_ (cleaved at R320 only) were drastically slower (Fig. 4*A*). The slower loss of FRET in proT_RS,R271Q_ is due to a conformational change that coincides with cleavage at R320 and formation of meizothrombin (Fig. 4*E*). These results demonstrate that activation of proT_RS_ by fXa proceeds along the prethrombin-2 pathway and that the loss of FRET is caused by cleavage at R271.

Next we replaced the P11–P8′ residues at the R271 site with those at the R320 site in the proT_RS_ background, proT_RS,320/320_. Two additional mutants contained replacement of the nonprimed P11–P1 residues, proT_RS,320/320np_, or the primed P1′–P8′ residues, proT_RS,320/320p_ (Fig. 1*B*). We verified that the single-molecule FRET (smFRET) profiles of these mutants populate the same compact conformation as wild-type prothrombin (Fig. 4*E*), ruling out effects on the overall architecture of the protein (data not shown). Activation by fXa monitored by changes in FRET showed a 9-fold reduction in the *k*_cat_/*K*_m_ value for proT_RS,320/320_ compared with proT_RS_ (Table 1, Fig. 4*C*), in agreement with a delayed appearance of the prethrombin-2 band by SDS-PAGE (Fig. 2). The loss of specificity in proT_RS,320/320_ is due mostly to replacement of the nonprimed residues: the *k*_cat_/*K*_m_ value was reduced ninefold for proT_RS,320/320np_ but only marginally for proT_RS,320/320p_ (Table 1, Fig. 4*C*). The primed and nonprimed portions of the sequence contribute to cleavage by fXa in an additive manner, with a free energy of coupling Δ*G*_c_ = –0.2 kcal/mol (31), underscoring a nearly independent contribution of the two fragments. Because the P1, P2, and P4 residues are conserved between the R271 and R320 sites and the P3 residue is acidic in both cases, our data show that the distal residues that occupy the P5–P11 positions play an important role for the preferential recognition of the R271 site by fXa.

**Table 1:** Specificity constants (*k*_cat_/*K*_m_ in μM^−1^s^−1^) for cleavage of R271 in ProT_RS_ (by fXa) and Mz_RS_ (by prothrombinase) measured by loss of FRET

Sequence at R271	ProT_RS_	Mz_RS_
WT	0.53 ± 0.02	166 ± 2
P11–P8′ of the R320 site	0.058 ± 0.002	9.5 ± 0.1
P11–P1 of the R320 site	0.061 ± 0.001	117 ± 8
P1′–P8′ of the R320 site	0.33 ± 0.01	127 ± 9

Experimental conditions: 20 mM Tris, 145 mM NaCl, 5 mM CaCl_2_, 50 μM phospholipids, 0.1% PEG8000, 0.01% Tween20, pH 7.5 at 20 °C.

### FRET analysis of prothrombin activation by prothrombinase

FRET measurements were extended to prothrombin activation by prothrombinase along the meizothrombin pathway triggered by cleavage at R271. Monitoring cleavage at this site in prothrombin by loss of FRET is challenging. The initial cleavage of prothrombinase at R320 causes a partial loss of FRET due to a conformational change, while the subsequent cleavage at R271 leads to complete loss of FRET due to separation of the protease domain from the rest of the molecule (Fig. 4*E*). Therefore, cleavage at R271 was studied with the meizothrombin version of proT_RS_, *i.e.*, Mz_RS_, that was similarly labeled with the Alexa Flour 555/647 pair at kringle 1 and the protease domain. Meizothrombin becomes a new substrate for prothrombinase after the initial cleavage of prothrombin at R320. Furthermore, cleavage of meizothrombin only takes place at R271, thereby making assignment of any loss of FRET unambiguous.

The progress curve of Mz_RS_ activation by prothrombinase obeyed a single exponential (Fig. 4*B*) with a rate of 166 μM^−1^s^−1^, in agreement with the value of *k*_cat_/*K*_m_ = 230 μM^−1^s^−1^ estimated for cleavage of R271 in meizothrombin (9). A control run with the mutant Mz_RS,R271Q_ resulted in no FRET changes (Fig. 4*B*), as expected. The additional meizothrombin mutants Mz_RS,320/320_, Mz_RS,320/320np_, and Mz_RS,320/320p_ were constructed and characterized to study the effect of replacing the P11–P8′ sequence at the R271 site, in full or in terms of its nonprimed and primed portions, as done with prothrombin (Fig. 1*B*). The smFRET profiles of these meizothrombin constructs were identical with the open form populated by Mz_RS_ (Fig. 4*E*), with no evidence of specific effects of the mutations on the open conformation (data not shown). The rate of activation of Mz_RS,320/320_ was 17-fold slower than that of Mz_RS_ (Table 1, Fig. 4*D*), in agreement with the delayed appearance of the fragment F1.2 band that was observed by SDS PAGE (Fig. 2). Hence, the sequence at the R320 site does not drive preferential cleavage by prothrombinase. If so, the rate of activation of Mz_RS,320/320_ would be faster than that of Mz_RS_. Furthermore, both Mz_RS,320/320np_ and Mz_RS,320/320p_ were activated at the same rate as Mz_RS_ (Table 1, Fig. 4*D*), indicating strong coupling (Δ*G*_c_ = 1.3 kcal/mol) between the two segments in reducing interaction with prothrombinase. Mutations introduced in the entire P11–P8′ prove that the primed and nonprimed residues act independently for the activation of prothrombin by fXa, but are strongly coupled when meizothrombin is activated by prothrombinase. The transition from the closed to the open form likely changes the cross talk between nonprimed and primed segments of the activation site at R271.

### smFRET studies of the site at R271

Our results indicate that the preference of prothrombinase for cleavage at R320 is specific to the position rather than the sequence. The specificity may arise from the components of the prothrombinase complex, fXa and/or fVa, blocking access to the site at R271. In this case, a conformational change of prothrombin and/or prothrombinase must take place to dock R271 into the active site of fXa. We have shown that activation of prothrombin produces a transition from the closed to open form (2, 23, 24) with significant elongation of the protein (Fig. 4*E*). Whether such transition affects the environment of R271 has not been established. To that end, smFRET studies were carried out with Alexa Flour 555/647 pairs introduced with Cys substitutions of R271 and a second residue in kringle 1 (S101C/R271C), kringle 2 (S210C/R271C), or the protease domain (Q576C/R271C). A fourth mutant labeled at two sites in the protease domain (Q576C/S478C) served as control. In all cases, the S525A substitution was added to prevent (auto)proteolysis when converting prothrombin to meizothrombin.

**Figure 5:**
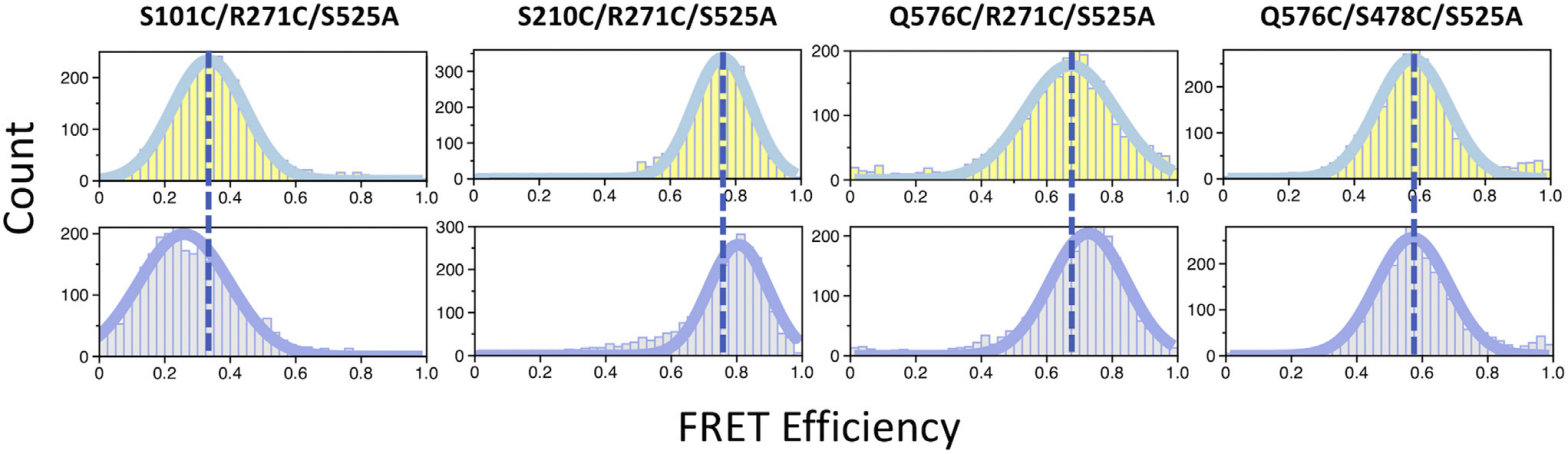
**smFRET studies.** smFRET profiles of prothrombin (*yellow*) and meizothrombin (*gray*) mutants labeled with the Alexa Flour 555/647 FRET pair at the indicated Cys residues. Experimental conditions are: 20 mM Tris, 145 mM NaCl, 5 mM CaCl_2_, 0.01% Tween, pH 7.5 at 20 °C.

smFRET profiles feature a single population of interprobe distances for all prothrombin variants (Fig. 5). Assuming an *R*_0_ = 51 Å for the Alexa Flour 555/647 pair, the calculated interprobe distances by FRET are 57 Å for S101C/R271C, 42 Å for S210C/R271C, 45 Å for Q576C/R271C, and 47 Å for Q576C/S478C. These values are in excellent agreement with the Cα-Cα distances measured from the crystal structure of prothrombin in the closed form (PDB ID: 6C2W) (2), *i.e.*, 53 Å for S101-R271, 45 Å for S210-R271, 46 Å for Q576-R271, and 42 Å for Q576-S478. When the prothrombin mutants were converted to meizothrombin, a change was observed in the efficiency profile of all constructs labeled at the 271 site. The population shifted to lower FRET efficiency indicating longer distance for the S101C/R271C pair, but to higher FRET efficiency indicating shorter distance for the S210C/R271C and Q576C/R271C pairs (Fig. 5). No change was observed for the Q576C/S478C pair, proving that the change observed in the Q576C/R271C pair upon formation of meizothrombin is due to movement of R271, which is also detected by the other S101C/R271C and S210C/R271C pairs (Fig. 5). Hence, the segment hosting the site of cleavage at R271 changes its conformation when the closed form of prothrombin transitions to the open form of meizothrombin.

## Discussion

Among the two possible sites of cleavage of prothrombin at R320 and R271, prothrombinase has a strong preference for R320 (6, 9, 11, 12). The cleavage generates the active intermediate meizothrombin on the pathway of activation to the final product thrombin and ensures immediate activity necessary to respond to vascular injury. Possible explanations for this preferential cleavage include more favorable docking of the R320 site into the active site of fXa in the prothrombinase complex predicted by molecular modeling (32), kinetics of activation interpreted in terms of distinct conformations of prothrombinase (9, 33) or prothrombin (8, 26). The recently identified open-closed equilibrium of prothrombin links preferential cleavage at R320 to the closed form and preferential cleavage at R271 to the open form (2, 22, 24). None of these previous explanations has addressed the role of the sequence at the sites of cleavage, perhaps because of high conservation of the critical P1–P4 residues at the two sites (Fig. 1). However, although the P1–P4 residues are critical for recognition by trypsin-like proteases either through independent or coupled interactions (34, 35), the energetics of binding of macromolecular substrates like prothrombin likely depend on more extended portions of the sequence at the cleavage site.

Our findings show that in the absence of fVa, the specificity of fXa for cleavage at R271 and activation along the prethrombin-2 pathway are strongly influenced by the sequence of amino acids that surround this site. The proT_271/271_ mutant, engineered by replacing the P11–P8′ sequence at the R320 site with that of the R271 site, is simultaneously activated by fXa along the prethrombin-2 and meizothrombin pathways. The reverse replacement in the proT_320/320_ mutant results in slower cleavage at the R271 site. The observation that sequence governs the kinetic preference of bond cleavage in prothrombin is similar to the inactivation of factor VIIIa by activated protein C (APC). This reaction entails proteolytic cleavage at two sites, R336 and R562, with the former cleaved by APC at a faster rate (36, 37). Replacing the P4–P3′ residues at the R336 site with those of the R562 site produces a drastic reduction in the rate of cleavage at the swapped site, while the reverse replacement enhances the rate of cleavage at the less specific site (36, 37).

In contrast to the reaction with fXa, our data show that the preference for cleavage at R320 by prothrombinase is specific to the site and cannot be transferred to the other regions of prothrombin. Replacing the P11–P8′ residues at the R271 site with those of the R320 site, and vice versa, does not change the preference of prothrombinase for cleavage at the R320 site first and leaves activation along the meizothrombin pathway intact. This proves that the dominant closed form of prothrombin (2, 22) provides a best fit with prothrombinase through the P11–P8′ residues at the R320 site, but only when these residues occupy the 310–328 sequence of the zymogen. Steric blockage of R271 likely makes R320 the only available cleavage site for prothrombinase, and this may well be a major role played by fVa in the prothrombinase complex. After cleavage at R320, prothrombin transitions to the active intermediate meizothrombin that is also a substrate of prothrombinase. A conformational change is needed to expose the site at R271 presumably blocked by fVa. Our smFRET data reveal that formation of meizothrombin produces a significant structural change that also affects the environment of R271 (Figs. 4 and 5). The transition of meizothrombin from the closed to open form (2, 24) may suffice to relocate R271 into the active site of fXa, but additional conformational changes may involve fVa and fXa. Hence, the molecular surface of interaction and the conformation of prothrombinase likely change when prothrombin is replaced by meizothrombin as a substrate. Validation of this scenario and of previous proposals (8, 9, 26, 32, 33) will come from structures of prothrombin and meizothrombin bound to prothrombinase, as a segue to the recent cryo-EM structures of fV and fVa (38).

## Experimental procedures

### Protein expression and purification

A quick change lightning site-directed mutagenesis kit (Agilent Technologies) was used to introduce substitutions into a plasmid expressing the human prothrombin gene carrying a C-terminal HPC-4 tag. Transfection of baby hamster kidney cells and selection of stably expressing clones were done as described for protein C (39). Prothrombin secreted in media (6–8 L) collected from CellSTACK culture factories with ten chambers (Corning) was initially purified by immunoaffinity chromatography (40). After diluting the [NaCl] < 40 mM, prothrombin was loaded onto a 1 ml Q-sepharose column (Cytiva) attached to the bottom of a 1 ml Heparin column (Cytiva) equilibrated with 20 mM Tris, pH 7.5, 40 mM NaCl, and 10 mM EDTA. The heparin column was detached and prothrombin was eluted from the Q-sepharose column using 0.04–1 M NaCl gradient. Protein purity was further refined on a superdex 200 size-exclusion column (Cytiva).

### smFRET studies

Prothrombin was labeled at the engineered Cys residues with Alexa Flour 555-C2-maleamide and Alexa Flour 647-C2-maleamide (Invitrogen) according to an established protocol (23). Prothrombin (14 μM) was incubated for 1 h with 2.8-fold molar excess dithiothreitol (DTT) in a labeling buffer composed of 20 mM Tris, 350 mM NaCl, pH 7.5. Excess DTT was removed on a Zeba spin desalting column (ThermoFisher) equilibrated with labeling buffer, after which the protein (12 μM) was incubated in the dark with 2.5-fold molar excess Alexa Fluor 555-C2-maleamide and 2.5-fold molar excess Alexa Fluor 647-C2-maleamide for 2 h. A size-exclusion superdex 200 column (Cytiva) equilibrated with labeling buffer was used to separate unreacted fluorophores from the labeled protein.

Labeled prothrombin (1.5 μM) was converted into meizothrombin upon incubation for 35 min with 0.025 units ecarin (Sigma-Aldrich) at 37 °C. Completion of the activation process during incubation was confirmed with SDS-PAGE analyses. Once activated, meizothrombin was diluted by a factor of 10,000 and immediately used for smFRET measurements of freely diffusing proteins using a MicroTime 200 confocal microscope equipped with pulsed interleaved excitation (PIE). All measurements were collected using 150 pM of protein in 20 mM Tris, 145 mM NaCl, 5 mM CaCl_2_, 0.01% Tween 20, at pH 7.5, and 20 °C. Data were initially processed with the PIE analysis with MATLAB (PAM) software (41). Bursts with more than 35 photons were initially integrated into 0.5 ms time bins. After applying the correct γ-factor and correction factors for donor leakage and direct acceptor excitation, histograms were constructed from molecules with labeling stoichiometry in the 0.3–0.7 range. The selection of molecules with stoichiometry in this range ensured that donor-only (S ∼ 1) and acceptor-only (S ∼ 0–0.25) labeled molecules were excluded from the analyses. An ALEX 2CDE cutoff <25 was also applied to filter donor or acceptor blinking and bleaching events (41). Data were then exported into Origin 8.1 and fitted using Gaussian functions.

### SDS-PAGE and FRET kinetics

Prothrombin (2 μM) was reacted with either 0.2 nM or 4 nM human fXa in the presence or absence of 30 nM human fVa (Haematologic Technologies) under experimental conditions: 20 mM Tris, 145 mM NaCl, 5 mM CaCl_2_, 60 μM phosphatidylcholine:phosphatidylserine (PC:PS) [3:1], 60 μM DAPA, and 0.1% PEG8000 at pH 7.5. At different time points, the activation was quenched by adding NuPAGE LDS loading buffer mixed with 10% (vol/vol) 2-mercaptoethanol and 10 mM EDTA. After boiling for 4 min, quenched samples containing ∼1.5 μM of prothrombin were analyzed by SDS-PAGE. Optical densitometry was performed using the ImageJ software.

The FRET assay was conducted using proT_RS_ (see Results) variants labeled at the 101/478 positions with Alexa Flour 555-C2-maleamide and Alexa Flour 647-C2-maleamide as described (23). proT_RS_ constructs (1.5 μM) were activated to the corresponding Mz_RS_ constructs by incubation for 35 min with 0.0125 units ecarin at 37 °C and immediately used for the kinetic measurements after dilution. SDS-PAGE confirmed complete activation of prothrombin by ecarin without additional cleavages.

A Horiba Flouromax-4 spectrofluorometer was used to continuously monitor the loss of acceptor fluorescence emission at *λ*_em_ = 660 nm upon donor excitation at 540 nm, with the excitation and emission slit widths kept at 8.5 nm. All reactions were monitored in a cuvette with 0.3 cm pathlength, and the temperature in the cell holder was regulated with a circulating water bath. FRET measurements were performed under pseudo-first-order conditions where the concentration of prothrombin and meizothrombin constructs (10 nM each) was maintained below the *K*_m_. Reactions were initiated upon mixing the substrate with either human factor Xa (Enzyme Research Laboratories) or prothrombinase under experimental conditions: 20 mM Tris, 145 mM NaCl, 5 mM CaCl_2_, 50 μM PC:PS [3:1], 0.01% Tween20, and 0.1% PEG8000, pH 7.5 at 20 °C. Blank measurements containing all reaction components except the enzyme were carried out for each substrate used. The progress curve corrected for blank subtraction was fitted to a single exponential, and the specificity constant was obtained by dividing the *k*_obs_ by the enzyme concentration. All measurements were performed in triplicates.

### Coupling free energy

The coupling free energy between two mutational events, such as the replacement of primed and nonprimed residues of the sequence at the site of cleavage, can be quantified using the expression (31)

(1) $$\Delta G_{c}=-RTln\frac{s_{wt}s_{12}}{s_{1}s_{2}}$$

where *R* is the gas constant, *T* is the absolute temperature, and *s* refers to the *k*_cat_/*K*_m_ of wild-type, $s_{wt}$, the two singly substituted constructs $s_{1}$ and $s_{2}$, and the doubly substituted construct $s_{12}$. In the case of prothrombin and meizothrombin, these constructs refer to replacement of the P11–P1 nonprimed, P1′–P8′ primed, and entire P11–P8′ sequence of the R271 site. A value of $\Delta G_{c}=0$ indicates that the individual substitutions make additive or independent contributions to the specificity of the double-substituted construct. A value of $\Delta G_{c}\neq$0 indicates that substitutions in the primed and nonprimed residues make nonadditive or coupled contributions to the specificity of the fully substituted sequence that either *reduces* $\Delta G_{c}>0$ or *enhances* $\Delta G_{c}<0$.

## Data availability

All data described in the manuscript are contained within the manuscript.

## Conflict of interest

The authors declare that they have no conflicts of interest with the contents of this article.
